# Comprehensive Comparison of Multiple-Detector Computed Tomography and Dynamic Magnetic Resonance Imaging in the Diagnosis of Hepatocellular Carcinoma with Varying Degrees of Fibrosis

**DOI:** 10.1371/journal.pone.0166157

**Published:** 2016-11-09

**Authors:** Ming-Tsung Lin, Chih-Chi Wang, Yu-Fan Cheng, Hock-Liew Eng, Yi-Hao Yen, Ming-Chao Tsai, Po-Lin Tseng, Kuo-Chin Chang, Cheng-Kun Wu, Tsung-Hui Hu

**Affiliations:** 1 Division of Hepato-Gastroenterology, Department of Internal Medicine, Kaohsiung Chang Gung Memorial Hospital, Kaohsiung, Taiwan; 2 Department of Surgery, Kaohsiung Chang Gung Memorial Hospital, Kaohsiung, Taiwan; 3 Department of Radiology, Kaohsiung Chang Gung Memorial Hospital, Kaohsiung, Taiwan; 4 Department of Pathology, Kaohsiung Chang Gung Memorial Hospital, Kaohsiung, Taiwan; 5 Graduate Institute of Clinical Medical Sciences, College of Medicine, Chang Gung University, Taoyuan City, Taiwan; National Yang-Ming University, TAIWAN

## Abstract

**Background & Aims:**

Liver computed tomography and dynamic magnetic resonance imaging play an important role in the early detection of hepatocellular carcinoma. However, the American Association for the Study of Liver Diseases (AASLD) recommend the use of applied imaging studies for HCC diagnosis only in cirrhotic patients. This study aimed to comprehensively compare liver CT and dynamic MRI for HCC diagnosis before surgical resection over years in clinical practice, and also to compare the diagnostic differences between liver CT and dynamic MRI in HCCs with varying degrees of fibrosis.

**Methods:**

841 patients with liver tumor who had liver CT or dynamic MRI examinations followed by surgical resection were included in the study. We defined typical HCC imaging characteristics as early enhancement in the artery phase and early washout in the venous phase. The tumor size was recorded based on pathological examination after surgery. The pathologic fibrosis score was verified by the METAVIR scoring classification.

**Results:**

Among the 841 patients, 756 underwent liver CT and 204 underwent dynamic liver MRI before surgery. The etiologies of chronic liver disease included hepatitis B virus, hepatitis C virus, hepatitis B and C virus, and non-hepatitis B or C virus. The sensitivity and accuracy of liver CT or MRI for HCC diagnosis was approximately 80%~90%. Liver CT had a diagnostic accuracy for HCC similar to that of dynamic MRI, and liver fibrosis stage did not influence their diagnostic efficacies.

**Conclusions:**

The application of 4-phase dynamic CT and MRI exhibit similar diagnostic accuracy for hepatocellular carcinoma, in tumors of sizes 1 to 2 cm and >2 cm. Liver fibrosis status did not affect the diagnostic accuracy of liver CT or MRI for HCC. The AASLD and EASL restrictions of dynamic imaging studies for HCC diagnosis to cirrhotic patients alone are unnecessary.

## Introduction

Hepatocellular carcinoma (HCC) is one of the most common cancers and one of the leading causes of cancer-related death worldwide [1]. The incidence of HCC varies geographically because the major causative factors are different in different countries [2]. However, the greatest risk factor for development of HCC is a background of liver cirrhosis [3]. If diagnosed in its early stages, HCC can be potentially cured. As a result, patients at high risk of developing HCC should be enrolled in surveillance programs [4]. Application of diagnostic imaging is important in the screening, diagnosis, and therapy of HCC. With advancements in liver computed tomography (CT) and magnetic resonance (MR) scanners, imaging studies have emerged as important modalities for assessing cirrhosis and HCC. The typical radiological presentation of HCC is the phenomenon of arterial vascularity and venous washout [5–6]. In the arterial phase of a liver CT scan or MR imaging (MRI), the HCC tissue has a brighter signal than the surrounding liver, and in the venous or delayed phase of the study, the lesion is less enhanced than the surrounding liver [4,7–8]. The challenge is to distinguish small HCCs from regenerative or dysplastic nodules 1–2 cm in diameter [8]. Early and smaller HCCs, unlike classical HCCs, are hypovascular in dynamic imaging studies, because there is a reduction in portal venous supply, but arterial vascularization is not fully developed [9–11]. Guidelines released by the European Association for the Study of the Liver (EASL) in 2001 and by the American Association for the Study of Liver Diseases (AASLD) in 2005 and 2010 all addressed the issue of HCC diagnosis [4,12–13]. The latest AASLD guidelines (2010) specify that if the appearance is typical for HCC (size >1 cm) in 4-phase liver CT or dynamic liver MRI (i.e., the lesion shows hypervascularization in the arterial phase with early washout in the portal venous or the equilibrium phase), a biopsy is unnecessary and the lesion can be treated as HCC. However, if the vascular profile on imaging is not characteristic or if the nodule is detected in a non-cirrhotic liver, biopsy should be performed [4]. Histological confirmation plays an important role in the diagnosis of nodules smaller than 2 cm when imaging appearances are not typical [10,14]. However, accurate needle placement is difficult and implantation of HCCs into the tract, or seeding, has been reported [14,15]. The diagnostic sensitivity of alpha-fetoprotein (AFP) for HCC is low, and it is no longer recommended as a diagnostic modality in the 2010 AASLD guidelines [4]. However, an abnormal finding in the surveillance ultrasound or higher AFP levels warrant dynamic liver MRI or CT for HCC diagnosis. CT scanning is commonly used to assess tumors in the liver. Indeed, multi-detector liver CT has the advantage of fast, high-quality, and thin-section imaging. Moreover, in a CT examination, patients can be examined in a short time and can easily tolerate the shorter duration of breath-holding. The stage of cancer determines the therapeutic choice. As a result, the purpose of surveillance is to identify HCC at an early stage when a cure is possible. Early diagnosis of HCC is extremely important in improving the survival of patients. In this study, we retrospectively analyzed and compared the sensitivity, specificity, positive predictive value (PPV), negative predictive value (NPV), and accuracy of liver CT and MRI in the diagnosis of HCC and tested their application in varying degrees of liver fibrosis. To our knowledge, this is the first report that correlates liver pathology and fibrosis with imaging studies for HCC diagnosis in explanted liver.

## Materials and Methods

The study protocol conformed to the ethical guidelines of the Declaration of Helsinki and was approved by the institutional review board in 2011 in Kaohsiung Chang Gung Memorial Hospital. Informed consent was waived for this retrospective study. Between January 2006 and October 2010, 1016 patients underwent liver tumor resections or liver transplantation in the Chang Gang Memorial Hospital, Kaohsiung, Taiwan. Of these, 841 patients underwent liver CT or MRI examinations or had a pathological fibrosis score analysis, and were therefore enrolled in this study. The exclusion criteria were patients who did not undergo liver CT or MRI examination before surgery, did not have a pathological fibrosis score analysis, or did not have liver tumors in the explanted liver. We defined the typical HCC imaging characteristics as early enhancement in the arterial phase and early washout in the venous phase. A variety of lesions such as dysplastic nodules, arterioportal shunts, and hemangiomas can manifest with increased arterial enhancement similar to HCC [16–18]. As a result, arterial enhancement alone without venous washout was not considered a typical HCC imaging characteristic. Histological and surgical reports were reviewed to confirm HCC or other liver tumors (including hemangioma, cholangiocarcinoma, and other metastatic tumors). In order to avoid confusion with other smaller regenerative nodules and to obtain more accurate data, we only took the largest hepatic tumor for analysis. The tumor size was recorded based on the pathology report after surgery. Four-phase liver CT or dynamic liver MRI images were read by radiologists with extensive experience in liver and HCC imaging. In addition, pathological results were read by pathologists with sufficient experience in the field and who were blinded to the clinical and radiological results. Pathologic fibrosis scores were verified using the METAVIR score classification [19]. In the METAVIR fibrotic score classification, a fibrosis score of 0 indicates no fibrosis, 1 indicates portal fibrosis without septa, 2 indicates portal fibrosis with a few septa, 3 indicates numerous septa without cirrhosis, and 4 indicates cirrhosis.

### Computed tomography imaging technique

Computed tomography examinations were performed using a helical CT (Toshiba, 120KVP) with a 4-phase (non-contrast, arterial, portal and delayed phases) technique. The scans through the liver were obtained in a clockwise direction, from the lower chest to the liver inferior edge in 5-mm contiguous sections. Approximately 80 mL of contrast medium was injected at 2 mL/sec, and the arterial phase scan was started 30 sec after the initiation of injection. The portal phase scan was performed 20 sec after the end of the arterial phase, and the venous phase scan was performed 20 sec after the end of the portal phase.

### Magnetic resonance imaging technique

All MRI examinations were performed using a 1.5-T MR system (Philips, Amsterdam, The Netherlands). The contrast medium used was intravenous gadolinium diethylenetriamine pentaacetic acid (Gd-DTPA), at a dose of 0.2 mg/kg and a rate of 1.6–1.8 mL/sec. Pulse sequences included T1WI, T2WI, T2WI Fsat, heavy T2WI, long T2WI, and enhanced T1WI, which included 3 phases, the first of which was obtained 15 sec after IV infusion of Gd-DTPA, while the second and third were obtained after 30-sec intervals. All sequences were obtained at 8 mm thickness and 2-mm gap for the whole liver.

### Statistical analysis

Patients with typical HCC features observed in liver CT or MRI, and those with nodules who did not exhibit typical HCC features in liver CT or MRI, and with varying degrees of liver fibrosis, were compared using χ^2^ test or Fisher’s exact test for categorical variables. A *P* value of < 0.05 was considered statistically significant.

## Results

From January 2006 to October 2010, we enrolled 841 patients with liver tumors who underwent tumor resection or liver transplantation. Among the 841 patients, 756 underwent liver CT study and 204 underwent dynamic liver MRI study before surgery. The demographic and clinical characteristics of patients who underwent liver CT are as follows: male/female ratio, 555/201; mean age, 55.81 years; mean tumor size, 5.44 cm; liver tumor size of 1–2 cm, 131 patients; and liver tumor size >2 cm, 625 patients (Table 1). The etiologies of chronic liver disease in the liver CT group included hepatitis B virus (n = 374), hepatitis C virus (n = 157), hepatitis B and C virus (n = 22), and non-hepatitis B or C virus (n = 202). The demographic and clinical characteristics of patients who underwent dynamic liver MRI are as follows: male/female ratio, 142/62; mean age, 54 years; mean tumor size, 4.04 cm; liver tumor size of 1–2 cm, 58 patients; and liver tumor size >2 cm,146 patients. The etiologies of chronic liver disease in the dynamic MRI group included hepatitis B virus (n = 96), hepatitis C virus (n = 36), hepatitis B and C virus (n = 6), and non-hepatitis B or C virus (n = 65). The overall sensitivity, specificity, PPV, NPV, and accuracy of 4-phase liver CT for HCC diagnosis were 87.5%, 76.3%, 92.6%, 64.4%, and 84.9%, respectively (Table 2). The overall sensitivity, specificity, PPV, NPV, and accuracy of dynamic liver MRI were 83.6%, 77.1%, 87.5%, 71.1%, and 81.4%, respectively. There was no statistically significant difference in the HCC diagnostic value of liver CT and dynamic liver MRI (all *P* values >0.05). We compared both imaging techniques in detail based on tumor size. In liver tumors of 1–2 cm in size, the sensitivity, specificity, PPV, NPV, and accuracy of 4-phase liver CT for HCC diagnosis were 81.6%, 71.4%, 91.3%, 51.3%, and 79.4%, respectively (Table 3). Similarly, the sensitivity, specificity, PPV, NPV, and accuracy of dynamic liver MRI for 1–2 cm HCC tumor diagnosis were 81.4%, 60%, 85.4%, 52.9%, and 75.9%, respectively. There was no statistically significant difference in the diagnostic value of liver CT and dynamic liver MRI for 1–2 cm HCC tumors (all *P* values >0.05). In liver tumors >2 cm, the sensitivity, specificity, PPV, NPV, and accuracy of 4-phase liver CT for HCC diagnosis were 88.8%, 77.2%, 92.8%, 67.5%, and 86.1%, respectively (Table 4). The sensitivity, specificity, PPV, NPV, and accuracy of dynamic liver MRI for diagnosis of HCC tumors >2 cm were 84.6%, 81.8%, 88.5%, 76.3%, and 83.6%, respectively. The diagnostic efficiencies of liver CT and dynamic liver MRI for HCC tumors >2 cm were similar (all *P* values >0.05). Liver CT had similar sensitivity, specificity, PPV, NPV, and accuracy among tumors with different stages of liver fibrosis. In tumors of size 1–2 cm, liver CT had similar diagnostic efficacy in fibrosis stage 4 (cirrhosis) versus stages 0~3 (non-cirrhosis), stages 3~4 versus stages 0~2, stages 2~4 versus stages 0~1, and stages 1~4 versus stage 0 (Table 5). However, liver CT had a better NPV in the fibrosis stage 0~3 (non-cirrhosis) group than in the stage 4 (cirrhosis) group (69.2% versus 15.4%, *P* = 0.002). Liver CT also had better sensitivity in the fibrosis stage 2~4 group than in the stage 0~1 group (84.8% versus 61.1%, *P* = 0.043). In tumors >2 cm in size, the liver CT had similar diagnostic efficacy in fibrosis stage 4 (cirrhosis) versus stage 0~3 (non-cirrhosis), stage 3~4 versus stage 0~2, stage 2~4 versus stage 0~1, and stage 1~4 versus stage 0 (Table 6). However, liver CT had better specificity and NPV in stage 0~3 (non-cirrhosis) group than in the stage 4 (cirrhosis) group (*P* < 0.05). On the other hand, liver CT had a higher PPV for the fibrosis stage 4 (cirrhosis) group than the stage 0~3 (non-cirrhosis) group (*P* < 0.05). Dynamic MRI also showed similar diagnostic efficacy for various fibrotic stages (Table 7 and Table 8). However, for liver tumors 1–2 cm in size, dynamic MRI had a better NPV for the fibrosis stage 0~3 (non-cirrhosis) group than for the stage 4 (cirrhosis) group (*P* < 0.05) (Table 7). In tumors >2 cm, dynamic MRI had a higher PPV for the fibrosis stage 4 (cirrhosis) group than for the stage 0~3 (non-cirrhosis) group, but showed a better NPV for the stage 0~3 group than for the stage 4 group (Table 8).

**Table 1:** Demographic and clinical characteristics of patients in the liver CT and dynamic liver MRI groups.

	Liver CT	Dynamic MRI	*P* value
Patient number	756	204	
Mean age (years)	55.81 ± 12.27	54.00 ± 12.49	0.062
Gender	555/201	142/62	0.280
(male/female)			
Mean tumor size	5.44 ± 4.12	4.04 ± 3.13	<0.001
(cm)			
Number of patients	131 (17.3%)	58 (28.4%)	<0.001
with tumors 1–2			
cm in size			
Number of patients	625 (82.7%)	146 (71.6%)	<0.001
with tumors >2 cm			
in size			
Number of patients	104 (13.8%)	21 (10.3%)	0.192
with fibrosis stage			
0			
Number of patients	88 (11.6%)	18 (8.8%)	0.255
with fibrosis stage			
1			
Number of patients	40 (5.3%)	2 (1%)	0.008
with fibrosis stage			
2			
Number of patients	77 (10.2%)	14 (6.9%)	0.151
with fibrosis stage			
3			
Number of patients	281 (37.2%)	90 (44.1%)	0.071
with fibrosis stage			
4 (cirrhosis)			
Non-Hepatitis B or	202	65	0.146
C			
Hepatitis B	374	96	0.541
Hepatitis C	157	36	0.324
Hepatitis B and C	22	6	0.981

**Table 2:** Comparison of CT and MRI for HCC diagnosis (overall).

	CT	MRI	*P* value
Sensitivity	510/583 (87.5%)	112/134 (83.6%)	0.230
Specificity	132/173 (76.3%)	54/70 (77.1%)	0.888
PPV	510/551 (92.6%)	112/128 (87.5%)	0.063
NPV	132/205 (64.4%)	54/76 (71.1%)	0.294
Accuracy	642/756 (84.9%)	166/204 (81.4%)	0.218

PPV, positive predictive value; NPV, negative predictive value

**Table 3:** Comparison of CT and MRI for HCC diagnosis (tumor size 1–2 cm).

	CT	MRI	*P* value
Sensitivity	84/103 (81.6%)	35/43 (81.4%)	0.982
Specificity	20/28 (71.4%)	9/15 (60%)	0.507
PPV	84/92 (91.3%)	35/41 (85.4%)	0.361
NPV	20/39 (51.3%)	9/17 (52.9%)	0.909
Accuracy	104/131 (79.4%)	44/58 (75.9%)	0.587

PPV, positive predictive value; NPV, negative predictive value

**Table 4:** Comparison of CT and MRI for HCC diagnosis (tumor size >2 cm).

	CT	MRI	*P* value
Sensitivity	426/480 (88.8%)	77/91 (84.6%)	0.264
Specificity	112/145 (77.2%)	45/55 (81.8%)	0.482
PPV	426/459 (92.8%)	77/87 (88.5%)	0.172
NPV	112/166(67.5%)	45/59 (76.3%)	0.206
Accuracy	538/625 (86.1%)	122/146 (83.6%)	0.435

PPV, positive predictive value; NPV, negative predictive value

**Table 5:** Comparison of the diagnostic efficacy of CT for HCC tumors of various liver parenchymal fibrosis stages (tumor size 1–2 cm).

	**Fibrosis 4**	**Fibrosis 0–3**	***P* value**
	**(cirrhosis)**	**(non-cirrhosis)**	
Sensitivity	54/65 (83.1%)	30/38(78.9%)	0.602
Specificity	2/5 (40%)	18/23 (78.3%)	0.123
PPV	54/57 (94.7%)	30/35 (85.7%)	0.251
NPV	2/13 (15.4%)	18/26 (69.2%)	0.002
Accuracy	56/70 (80%)	48/61 (78.7%)	0.853
	**Fibrosis 3–4**	**Fibrosis 0–2**	
Sensitivity	64/76 (84.2%)	14/21 (66.7%)	0.116
Specificity	3/6 (50%)	1/3 (33.3%)	1.000
PPV	64/67(95.5%)	14/16 (87.5%)	0.245
NPV	3/15 (20%)	1/8 (12.5%)	1.000
Accuracy	67/82 (81.7%)	15/24 (62.5%)	0.048
	**Fibrosis 2–4**	**Fibrosis 0–1**	
Sensitivity	67/79 (84.8%)	11/18 (61.1%)	0.043
Specificity	3/7 (42.9%)	1/2 (50%)	1.000
PPV	67/71 (94.4%)	11/12 (91.7%)	0.552
NPV	3/15 (20%)	1/8 (12.5%)	1.000
Accuracy	70/86 (81.4%)	12/20 (60%)	0.071
	**Fibrosis 1–4**	**Fibrosis 0**	
Sensitivity	73/90 (81.1%)	5/7 (71.4%)	0.620
Specificity	3/8 (37.5%)	1/1 (100%)	0.444
PPV	73/78 (93.6%)	5/5 (100%)	1.000
NPV	3/20 (15%)	1/3 (33.3%)	0.453
Accuracy	76/98 (77.6%)	6/8 (75%)	1.000

PPV, positive predictive value; NPV, negative predictive value

**Table 6:** Comparison of the diagnostic efficacy of CT for HCC tumors of various liver parenchymal fibrosis stages (tumor size >2 cm).

	**Fibrosis 4**	**Fibrosis 0–3**	***P* value**
	**(cirrhosis)**	**(non-cirrhosis)**	
Sensitivity	187/206 (90.8%)	239/274 (87.2%)	0.223
Specificity	5/10 (50%)	107/135 (79.3%)	0.048
PPV	187/192 (97.4%)	239/267 (89.5%)	0.001
NPV	5/24 (20.8%)	107/142 (75.4%)	<0.001
Accuracy	192/216 (88.9%)	346/409 (84.6%)	0.140
	**Fibrosis 3–4**	**Fibrosis 0–2**	
Sensitivity	236/262 (90.1%)	166/192 (86.5%)	0.232
Specificity	7/13 (53.8%)	13/16 (81.3%)	0.226
PPV	236/242 (97.5%)	166/169 (98.2%)	0.742
NPV	7/33 (21.2%)	13/39 (33.3%)	0.253
Accuracy	243/275 (88.4%)	179/208 (86.1%)	0.450
	**Fibrosis 2–4**	**Fibrosis 0–1**	
Sensitivity	267/298 (89.6%)	135/156 (86.5%)	0.331
Specificity	7/13 (53.8%)	13/16 (81.3%)	0.226
PPV	267/273 (97.8%)	135/138 (97.8%)	1.000
NPV	7/38 (18.4%)	13/34 (38.2%)	0.061
Accuracy	274/311 (88.1%)	148/172 (86%)	0.515
	**Fibrosis 1–4**	**Fibrosis 0**	
Sensitivity	328/369 (88.9%)	74/85 (87.1%)	0.633
Specificity	10/18 (55.6%)	10/11 (90.9%)	0.096
PPV	328/336 (97.6%)	74/75 (98.7%)	1.000
NPV	10/51 (19.6%)	10/21 (47.6%)	0.016
Accuracy	338/387 (87.3%)	84/96 (87.5%)	0.966

PPV, positive predictive value; NPV, negative predictive value

**Table 7:** Comparison of the diagnostic efficacy of MRI for HCC tumors of various liver parenchymal fibrosis stages (tumor size, 1–2cm).

	**Fibrosis 4**	**Fibrosis 0–3**	***P* value**
	**(cirrhosis)**	**(non-cirrhosis)**	
Sensitivity	30/37 (81.1%)	5/6 (83.3%)	1.000
Specificity	1/5 (20%)	8/10 (80%)	0.089
PPV	30/34 (88.2%)	5/7 (71.4%)	0.268
NPV	1/8 (12.5%)	8/9 (88.9%)	0.003
Accuracy	31/42 (73.8%)	13/16 (81.3%)	0.736
	**Fibrosis 3–4**	**Fibrosis 0–2**	
Sensitivity	32/39 (82.1%)	2/3 (66.7%)	0.479
Specificity	1/5 (20%)	1/1 (100%)	0.333
PPV	32/36 (88.9%)	2/2 (100%)	1.000
NPV	1/8 (12.5%)	1/2 (50%)	0.378
Accuracy	33/44 (75%)	3/4 (75%)	1.000
	**Fibrosis 2–4**	**Fibrosis 0–1**	
Sensitivity	32/39 (82.1%)	2/3 (66.7%)	0.479
Specificity	1/5 (20%)	1/1 (100%)	0.333
PPV	32/36 (88.9%)	2/2 (100%)	1.000
NPV	1/8 (12.5%)	1/2 (50%)	0.378
Accuracy	33/44 (75%)	3/4 (75%)	1.000
	**Fibrosis 1–4**	**Fibrosis 0**	
Sensitivity	33/41 (80.5%)	1/1 (100%)	1.000
Specificity	1/5 (20%)	1/1 (100%)	0.333
PPV	33/37 (89.2%)	1/1 (100%)	1.000
NPV	1/9 (11.1%)	1/1 (100%)	0.200
Accuracy	34/46 (73.9%)	2/2 (100%)	1.000

PPV, positive predictive value; NPV, negative predictive value

**Table 8:** Comparison of the diagnostic efficacy of MRI for HCC tumors of various liver parenchymal fibrosis stages (tumor size: >2cm).

	**Fibrosis 4**	**Fibrosis 0–3**	***P* value**
	**(cirrhosis)**	**(non-cirrhosis)**	
Sensitivity	43/48 (89.6%)	34/43 (79.1%)	0.165
Specificity	2/2 (100%)	43/53 (81.1%)	1.000
PPV	43/43 (100%)	34/44 (77.3%)	0.001
NPV	2/7 (28.6%)	43/52 (82.7%)	0.006
Accuracy	45/50 (90%)	77/96 (80.2%)	0.130
	**Fibrosis 3–4**	**Fibrosis 0–2**	
Sensitivity	52/58 (89.7%)	23/30 (76.7%)	0.122
Specificity	1/2 (50%)	5/7 (71.4%)	1.000
PPV	52/53 (98.1%)	23/25 (92%)	0.239
NPV	1/7 (14.3%)	5/12 (41.7%)	0.333
Accuracy	53/60 (88.3%)	28/37 (75.7%)	0.103
	**Fibrosis 2–4**	**Fibrosis 0–1**	
Sensitivity	54/60 (90%)	21/28 (75%)	0.104
Specificity	1/2 (50%)	5/7 (71.4%)	1.000
PPV	54/55 (98.2%)	21/23 (91.3%)	0.206
NPV	1/7 (14.3%)	5/12 (41.7%)	0.333
Accuracy	55/62 (88.7%)	26/35 (74.3%)	0.066
	**Fibrosis 1–4**	**Fibrosis 0**	
Sensitivity	65/74 (87.8%)	10/14 (71.4%)	0.210
Specificity	2/4 (50%)	4/5 (80%)	0.524
PPV	65/67 (97%)	10/11 (90.9%)	0.370
NPV	2/11 (18.2%)	4/8 (50%)	0.319
Accuracy	67/78 (85.9%)	14/19 (73.7%)	0.298

PPV, positive predictive value; NPV, negative predictive value

## Discussion

The most important risk factors for HCC are chronic hepatitis B, hepatitis C infection, and cirrhosis [12, 20, 21]. More than 80% of HCCs occur in patients with chronic liver disease or cirrhosis [22]. Most HCCs develop from a low-grade dysplastic nodule to a high-grade dysplastic nodule and finally to hypervascular HCC [23,24]. Imaging of the liver is an important technique in the detection and diagnosis of HCC. Due to surveillance programs for patients at high risk for the development of hepatocellular carcinoma, an increasing number of tumors are currently diagnosed at an early and asymptomatic stage [22]. As a result, several studies have reported that survival rates improved following surveillance [25–27]. The American Association for the Study of Liver Disease (AASLD) in 2010 emphasized the importance of dynamic imaging for HCC diagnosis [4]. CT is the most commonly used imaging technique to diagnose HCC. However, previous studies of the accuracy of different imaging modalities in the assessment of the hepatocellular carcinoma have revealed that spiral CT has a sensitivity of 67.5% and a specificity of 92.5%, compared to a sensitivity of 80.6% and a specificity of 84.8% for dynamic MRI [11]; MRI showed a significantly higher sensitivity for the identification of HCC than did spiral CT. Lee et al. have also reported a higher sensitivity for liver MRI than liver CT in a study of 78 patients [7]. However, in our study, based on the post-operative results of 841 patients, the sensitivity, specificity, positive PPV, NPV, and accuracy were similar between liver CT and MRI, in tumors 1–2 cm in size as well as in those >2 cm. No statistically significant difference was found between the 2 imaging modalities. The detection of small HCCs (1–2 cm), however, remains the most difficult challenge. A study by Furlan et al. reported that smaller HCCs exhibit less typical washout character in the venous or delayed phase [8]. In our study, the sensitivities of both liver CT and MRI were about 81.4% in liver tumors ranging from 1 to 2 cm in size, but their accuracies were 79.4% and 75.9%, respectively. No diagnostic difference was found between the 2 imaging modalities (*P* > 0.05). In liver tumors larger than 2 cm, the sensitivities of liver CT and MRI were 88.8% and 84.6%, respectively, and their accuracies were 86.1% and 83.6%, respectively. The sensitivity and accuracy of both liver CT and MRI were greater in tumors larger than 2 cm in size. Histologically, liver cirrhosis is characterized by fibrous septa and regenerative nodules. In a cirrhotic liver, early HCC can be difficult to distinguish from background nodularity. Tumor size is also an important factor for HCC diagnosis by imaging [9,28,29]. In the past, a correlation has been demonstrated between HCC grading and the size of the tumor in a surgical study [10]. Moreover, Iavarone et al. have also demonstrated that tumor grade influences the accuracy of imaging studies in the diagnosis of HCC [30]. In a study using multi-phasic contrast-enhanced CT, Sofue et al. showed that higher iodine concentration in the contrast material yielded a higher diagnostic sensitivity for detection of HCCs in cirrhotic liver [31]. Moreover, with progression from dysplastic nodules to HCCs, the blood supply shifts from portal venous to abnormal hepatic arterial supply, resulting in arterial enhancement [16, 32]. Smaller HCCs have less arterial blood supply, so that the nodule may appear hypovascular on liver CT or MRI [9–11]. As a result, the most important factors that influence the accuracy of imaging studies are tumor size and tumor grading, but not the liver fibrotic status. However, Tables 5, 6, 7 and 8 reveal mild diagnostic differences among various fibrotic stages. Most of these differences involve specificity, PPV, and NPV. However, this is a reasonable observation in clinical practice. In fact, if a liver tumor is not diagnosed as malignant using imaging studies or by clinical judgement prior to operation, the patient would not undergo liver transplantation or liver resection. As a result, the data might be skewed. However, in our sample set of 841 patients, only mild statistical differences in diagnostic efficacy were found among various fibrotic stages. Most of the comparisons showed similar results that were not statistically significant. The inevitable selection bias in clinical practice may play an important role in these statistical differences. The updated 2010 AASLD guidelines state that noninvasive diagnostic criteria can only be applied in cirrhotic or non-cirrhotic hepatitis B patients. Other non-cirrhotic patients are not eligible under these criteria. However, our comprehensive analysis involving a large number of patients who had liver tumors surgically resected or removed revealed that there is no significant difference in the diagnostic efficacy of liver CT or MRI among various stages of liver parenchymal fibrosis, in the presence or absence of cirrhosis. Liver fibrosis is not a decisive condition. In the past, some studies have evaluated the HCC diagnostic accuracy of CT or MRI. Kim et al. evaluated the accuracy of imaging studies and AFP for HCC diagnosis mostly by fine-needle biopsy [28], and concluded that liver CT can be used to diagnose HCCs >2 cm both in cirrhotic patients and in high-risk patients without cirrhosis. However, the possibility of false negatives or mistakes exists in the case of fine-needle biopsy. All 841 patients in our study underwent either liver resection or liver transplantation, allowing us to obtain the most accurate tumor size, and to accurately differentiate HCC from non-HCC lesions. Moreover, we were able to evaluate the liver parenchymal fibrosis score in detail. We found that the presence or absence of liver cirrhosis is not a decisive factor. Our previous published paper had validate the 2010 AASLD guideline and found that no matter hepatitis B or hepatitis C or non-hepatitis B and C had the similar diagnosis value for HCC in cirrhosis and non-cirrhosis patients [33]. Especially important of our present study, the latest AASLD guidelines (2010) defined that if the liver tumors (larger than 1 cm) with typical dynamic liver CT or MRI in cirrhosis patients, the HCC could be diagnosed without biopsy. Our comprehensive analysis proved the guideline legitimacy: the dynamic liver CT or MRI had the similar diagnostic efficacy for hepatocellular carcinoma diagnosis, no matter the fibrosis status. However, the present study had some limitations. Most important of all, this study was not prospective; consequently, there may have been a selection bias. However, this is an inevitable limitation due to the rare incidence of liver resections or transplantations if benign lesions were suspected prior to surgery in clinical practice.

In conclusion, 4-phase liver CT and dynamic liver MRI were both useful for distinguishing hypervascular HCCs from hepatic pseudolesions. MRI with dynamic gadolinium enhancement appears to have a similar diagnostic efficacy to helical CT for detecting HCCs. Our study demonstrated that liver CT is not inferior to dynamic MRI. The detection rates of both liver CT and MRI were higher for HCCs >2 cm than for HCCs <2 cm. According to our comprehensive liver fibrosis and imaging analysis, the presence or absence of cirrhosis had no influence on HCC diagnostic efficacy by liver CT or MRI. Therefore, the restrictions prescribed in the AASLD guidelines for HCC diagnosis are unnecessary.

## Supporting Information

S1 Dataset Minimal data of liver tumors post operation patients receiving liver CT or dynamic liver MRI in our program. Data Availability Statement: The authors uploaded minimal data of liver tumors post operation patients receiving liver CT or dynamic liver MRI in their program as supporting information named “S1 Dataset”. Detailed data about the study cohort is improper to provide online according to the privacy policy of Kaohsiung Chang Gung Memorial Hospital Ethics Committee. Readers who are interested in the study or who want to understand the authors' detailed data can contact them via the e-mail (Prof. Hu's e-mail address: dr.hu@msa.hinet.net).
(XLS)
